# Extended Spectrum Beta-Lactamase-Producing Gram-Negative Bacteria Recovered From an Amazonian Lake Near the City of Belém, Brazil

**DOI:** 10.3389/fmicb.2019.00364

**Published:** 2019-02-28

**Authors:** Dhara Y. Freitas, Susana Araújo, Adriana R. C. Folador, Rommel T. J. Ramos, Juliana S. N. Azevedo, Marta Tacão, Artur Silva, Isabel Henriques, Rafael A. Baraúna

**Affiliations:** ^1^Laboratory of Genomics and Bioinformatics, Center of Genomics and Systems Biology, Institute of Biological Sciences, Federal University of Pará, Belém, Brazil; ^2^Department of Biology and CESAM, University of Aveiro, Aveiro, Portugal; ^3^Federal Rural University of Amazon, Capanema, Brazil

**Keywords:** antibiotic resistance, *Escherichia coli*, *Acinetobacter baumannii*, *bla*_CTX–M_, whole genome analysis

## Abstract

Aquatic systems have been described as antibiotic resistance reservoirs, where water may act as a vehicle for the spread of resistant bacteria and resistance genes. We evaluated the occurrence and diversity of third generation cephalosporin-resistant gram-negative bacteria in a lake in the Amazonia region. This water is used for human activities, including consumption after appropriate treatment. Eighteen samples were obtained from six sites in October 2014. Water quality parameters were generally within the legislation limits. Thirty-three bacterial isolates were identified as *Escherichia* (*n* = 7 isolates), *Acinetobacter*, *Enterobacter*, and *Klebsiella* (*n* = 5 each), *Pseudomonas* (*n* = 4), *Shigella* (*n* = 3), and *Chromobacterium*, *Citrobacter*, *Leclercia*, *Phytobacter* (1 isolate each). Twenty nine out of 33 isolates (88%) were resistant to most beta-lactams, except carbapenems, and 88% (*n* = 29) were resistant to antibiotics included in at least three different classes. Among the beta-lactamase genes inspected, the *bla*_CTX–M_ was the most prevalent (*n* = 12 positive isolates), followed by *bla*_TEM_ (*n* = 5) and *bla*_SHV_ (*n* = 4). *bla*_CTX–M–15_ (*n* = 5), *bla*_CTX–M–14_ (*n* = 1) and *bla*_CTX–M–2_ (*n* = 1) variants were detected in conserved genomic contexts: *bla*_CTX–M–15_ flanked by IS*Ecp1* and Orf477; *bla*_CTX–M–14_ flanked by IS*Ecp1* and IS*903*; and *bla*_CTX–M–2_ associated to an ISCR element. For 4 strains the transfer of *bla*_CTX–M_ was confirmed by conjugation assays. Compared with the recipient, the transconjugants showed more than 500-fold increases in the MICs of cefotaxime and 16 to 32-fold increases in the MICs of ceftazidime. Two isolates (*Escherichia coli* APC43A and *Acinetobacter baumannii* APC25) were selected for whole genome analysis. APC43A was predicted as a *E. coli* pathogen of the high-risk clone ST471 and serotype O154:H18. *bla*_CTX–M–15_ as well as determinants related to efflux of antibiotics, were noted in APC43A genome. *A. baumannii* APC25 was susceptible to carbapenems and antibiotic resistance genes detected in its genome were intrinsic determinants (e.g., *bla*_OXA–208_ and *bla*_ADC–like_). The strain was not predicted as a human pathogen and belongs to a new sequence type. Operons related to metal resistance were predicted in both genomes as well as pathogenicity and resistance islands. Results suggest a high dissemination of ESBL-producing bacteria in Lake Água Preta which, although not presenting characteristics of a strongly impacted environment, contains multi-drug resistant pathogenic strains.

## Introduction

Bacterial resistance to antibiotics is currently one of the most serious public health concerns. The environment and particularly aquatic systems have been pointed as important reservoirs of resistance (Baquero et al., 2008; Taylor et al., 2011; Marti et al., 2014). These settings bring together indigenous bacterial communities and bacteria resulting from anthropogenic contamination, creating a milieu that may promote horizontal gene transfer (Pei and Gunsch, 2009; Jiao et al., 2017). Furthermore, significant quantities of contaminants accumulate in polluted aquatic systems and some of these contaminants were implicated in the selection of resistant bacteria (e.g., antibiotics, metals, disinfectants) (Henriques et al., 2016; Jiao et al., 2017). The environment was also confirmed as the origin of some of the most successfully widespread antibiotic resistance genes (e.g., *bla*_CTX–M_ and *bla*_OXA–48_; Poirel et al., 2002; Tacão et al., 2018). These evidences urgently ask to better understand the ecology of antibiotic resistance and the factors involved in resistance selection in aquatic systems. Dissemination of antibiotic resistance in these systems is particularly relevant when water is used for purposes that facilitate the transmission of bacteria to humans, namely for consumption, irrigation, recreational activities and fishing. Increasing our understanding of antibiotic resistance in specific aquatic systems is essential to suggest and implement mitigation strategies.

Nowadays, the spread of resistance to third generation cephalosporins in gram-negative bacteria is one of the major concerns in terms of antibiotic resistance. These antibiotics have great human health importance being often the first choice for the treatment of infectious diseases caused by gram-negative bacteria. Nevertheless, the levels of resistance to third generation cephalosporins have been increasing, and in several countries have reached levels that threaten their usefulness (WHO, 2014; ECDC, 2017). The most common and successful mechanism of resistance is the production of extended-spectrum beta-lactamases. According to a recent World Health Organization report, ESBL-producing Enterobacteriaceae are a critical human health concern (WHO, 2014). ESBLs can be classified into Ambler’s classes A (e.g., TEM, SHV, CTX-M, PER, VEB, GES) and D (OXA) (Ambler, 1980). Among these, enzymes of the CTX-M family are currently globally disseminated, often found in pathogenic bacteria of the family Enterobacteriaceae, and associated with mobile genetic elements (Bevan et al., 2017). In Brazil, CTX-M-producing bacteria have been frequently reported in hospital settings, with the most common variants being CTX-M-15 and CTX-M-2 (Rocha et al., 2016).

The problematic summarized above demands from the authorities measures to contain the spread of resistance to antibiotics. Aquatic environments may be one of the most important intervention areas. The occurrence of ESBL genes, including *bla*_CTX–M_, in different aquatic systems has been reported in several countries (Tacão et al., 2012; Zurfluh et al., 2013; Alves et al., 2014; Nascimento et al., 2017). In Brazilian aquatic systems, clinically relevant bacteria producing CTX-M enzymes have been recently described, e.g., in lakes (Nascimento et al., 2017), rivers (de Oliveira et al., 2017), wastewater (Dropa et al., 2016) and coastal water (Sellera et al., 2017). For the measures to be effective further studies are required to reveal which bacteria and which resistance and transfer mechanisms are present in these settings. There is a need to address different geographic areas, particularly ecologically relevant aquatic systems whose water is used for human activities.

In this work, we collected samples in an Amazonian lake. Water from this lake is used for water supply, irrigation and recreational activities (Santos et al., 2015). Gram-negative bacteria resistant to antibiotics were selected and mechanisms of resistance were characterized. The occurrence of genetic platforms that may contribute to multi-drug resistance in these bacteria (i.e., integrons) was also assessed. Two isolates belonging to species of public health interest (i.e., *Escherichia coli* and *Acinetobacter baumanii*) were selected for whole genome sequencing and analysis.

## Materials and Methods

### Sampling and Sample Analysis

**FIGURE 1:**
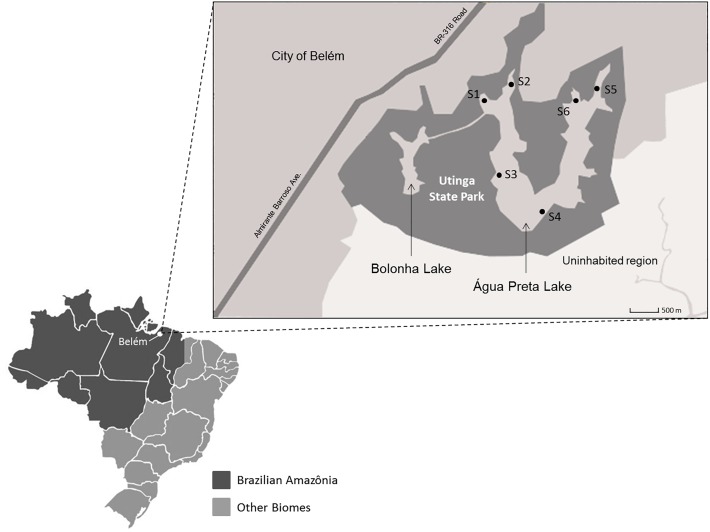
Map of the Utinga State Park (dark gray area). Sampling points are identified as S1, S2, S3, S4, S5, and S6. The urban area of Belém is represented by the gray area in the upper left half of the map. Therefore, the city is closer to the sampling points S1, S2, S5, and S6.

Lake Água Preta (1°25′7.849″S, 48°26′19.02″W) is an Amazonian mesotrophic lake located in the Utinga State Park, Pará, Brazil. It is located near a densely populated area that includes the city of Belém (population of approximately 1.5 million). This lake was chosen considering its importance in water supply, irrigation and recreational activities. It has great ecological relevance in the Amazonian area (Santos et al., 2015). The lake has a surface area of approximately 7 km^2^ and a maximum depth of 8.5 m. There are no relevant agricultural or livestock activities on the banks of the lake. There is, however, a record of untreated wastewater discharges resulting from a large number of illegal homes in the vicinity of the lake. Six sampling points were selected (Figure 1). One liter of water was collected in triplicate at each sampling point in October 2014. Samples were collected in 1 L polypropylene flasks, packed in an isothermal box with ice, and sent to the Faculty of Sanitary and Environmental Engineering laboratory, Federal University of Pará, Brazil. Water samples collected for microbiological analysis were stored in previously sterilized polypropylene flasks of 250 mL. Sampling and analytical methods were performed according to the procedures and recommendations described in Standards Methods for the Examination of Water and Wastewater (Rice et al., 2012). Physico-chemical parameters such as pH, conductivity, temperature, dissolved oxygen and salinity were analyzed at the sampling points by potentiometry using a multi-parametric probe (556 MPS; YSI, United States). The following parameters were determined by UV spectrophotometry (UV DR 2800; HACH, Germany): turbidity, total solids, true color, apparent color, total phosphorous, total nitrogen, total iron, chemical oxygen demand (COD), and the concentration of the ions nitrite, nitrate, ammonia, chloride, aluminum, manganese, nickel, cadmium, copper, zinc and sulfate. Biochemical oxygen demand (BOD) was determined using a manometric respirometric test in the equipment BODTrack II (HACH, United States). The Most Probable Number (MPN) of total coliforms and *E. coli* was determined using the chromogenic substrate Colilert 18/QUANTI-TRAY (IDEXX Laboratories, United States) according to the manufacturers’ protocol. Odor intensity was measured using sensorial panel, while alkalinity and acidity were determined by titrimetry.

Results were evaluated according to the resolution no. 357/2005 of the Environment National Council of Brazil (CONAMA, 2005).

### Bacteria Growth Conditions and Isolation

Water samples (1, 10, and 50 mL) were filtered through 0.45-μm-pore-size cellulose ester filters (Millipore). Membranes were placed onto MacConkey agar medium supplemented with cefotaxime (8 μg mL^−1^) (Sigma-Aldrich) and incubated at 37°C for 16 h. Individual colonies were purified in the same medium and stored in 20% glycerol at −80°C.

### DNA Extraction and Identification of the Isolates

For DNA extraction, the bacterial isolates were inoculated in Tryptic Soy Broth medium (Himedia) supplemented with cefotaxime (8 μg mL^−1^) and cultivated at 37°C overnight with aeration. An aliquot of 5 ml of the culture was centrifuged at 6,000 *g* at 4°C for 10 min. The cell pellet was subjected to DNA extraction using the DNeasy Blood and Tissue kit (Qiagen), according to the manufacturer’s protocol. The integrity of the DNA was visualized on 1% agarose gel. DNA was stored in TE buffer (Tris 10 mM, EDTA 1mM, pH 8.0) at −20°C.

To determine the phylogenetic affiliation of the isolates, the 16S rRNA gene was amplified using the universal primers 8F (5′-AGAGTTTGATCCTGGCTCAG-3′) and 1492R (5′-TACGGYTACCTTGTTACGACTT-3′). PCR was carried out in 50 μL reaction mixtures containing buffer 1×, 1.5 mM of MgCl_2_, 0.2 mM of dNTP, 0.2 pmol of each primer, 1 U of Taq DNA polymerase (Invitrogen) and 50–100 ng of DNA. Cycling conditions were as follows: an initial denaturation at 95°C for 5 min, followed by 35 cycles of 95°C for 1 min, 55°C for 1 min and 72°C for 1 min, and a final extension step of 72°C for 10 min. Amplicons were sequenced using the ABI 3730 DNA Analyzer platform (Thermo Fisher Scientific). Reverse and forward sequences were assembled with BioEdit v. 7.2.6.1 (Hall, 1999) and the consensus sequences (∼1.5 kb) were compared to the GenBank database using BLASTn^1^.

### Antibiotic Susceptibility Testing

To estimate the level of resistance of the isolates, the disk-diffusion method was used (Bauer et al., 1966). *E. coli* ATCC 25922 was used as quality control strain. Sixteen antibiotics were tested including amoxicillin (10 μg), amoxicillin + clavulanic acid (20–10 μg), ampicillin (10 μg), cephalotin (30 μg), cefotaxime (30 μg), ceftazidime (30 μg), cefepime (30 μg), imipenem (10 μg), aztreonam (30 μg), kanamycin (30 μg), gentamicin (10 μg), nalidixic acid (30 μg), ciprofloxacin (5 μg), chloramphenicol (30 μg), tetracycline (30 μg) and the combination of sulfamethoxazole + trimethoprim (25 μg). CLSI (2017) breakpoints were used to classify strains as susceptible, intermediate or resistant. Antibiotics were selected based on the CLSI guidelines, which specify the antibiotics that should be considered when characterizing Gram-negative non-fastidious organisms (e.g., Enterobacteriaceae, *Acinetobacter* spp. and *Pseudomonas aeruginosa*). Minimal inhibitory concentrations (MIC) were determined for cefotaxime and ceftazidime, following CLSI guidelines.

### PCR Amplification of Resistance Genes and Mobile Genetic Elements

Isolates were screened by PCR to determine the presence of genes conferring resistance to beta-lactams (*bla*_TEM_, *bla*_SHV_, *bla*_CTX–M_, *bla*_IMP_, *bla*_V IM_, *bla*_KPC_). We also analyzed the isolates for the presence of genes encoding integrases of class 1 (*intI1*) and 2 (*intI2*). The PCR reactions were performed in a GeneAmp PCR System 9700 (Applied Biosystem) using DNA purified as described above. PCR was carried out using buffer 1×, 1.5 mM of MgCl_2_, 0.2 mM of dNTP, 0.2 pmol of each primer and 1 U of Taq DNA polymerase (Invitrogen) with sufficient water for 25 μl of reaction. Primers used and PCR conditions were as previously described (Dallenne et al., 2010; Alves et al., 2014). The genomic context of *bla*_CTX–M_ was characterized by PCR-targeting IS*Ecp1*, IS*26*, orf477 and IS*903*, as previously described (Tacão et al., 2012). A negative and a positive control were included in each PCR experiment. The negative control differed from the reaction mixture by substituting DNA for the same volume of sterile dH_2_O. The amplicons were visualized on 1% agarose gels using the 1 kb Plus DNA ladder (Invitrogen) to assist in the identification of the PCR products.

### Mating Assays

Mating assays were performed for *bla*_CTX–M_-positive strains, as previously described (Moura et al., 2012). In short, donor strains and the rifampicin-resistant *E. coli* CV601 (recipient strain) were grown overnight in Luria–Bertani broth (LB) at 37°C, 180 rpm. Donor and recipient strains were mixed at a 1:1 ratio and centrifuged (5 min, 7,000 *g*) to precipitate cells. After discarding the supernatant, 1 mL of fresh LB was added and left overnight at 37°C, without shaking. Then, cells were centrifuged (5 min, 7,000 *g*) and resuspended in a 0.9% NaCl solution. Putative transconjugants were selected by plating 100 μL of this suspension in plate count agar (PCA) supplemented with rifampicin (100 μg/mL), and cefotaxime (8 μg/mL). To confirm the identity of the transconjugants we used BOX-PCR typing (Versalovic et al., 1994) and *bla*_CTX–M_ PCR amplification as described above.

### Genome Sequencing, Assembly and Analysis

Two multi-drug resistant isolates were selected randomly to represent phylogenetic groups with high clinical relevance (i.e., *Acinetobacter baumanii* and *E. coli*) and their genome was sequenced. Genomic DNA, extracted as described in Section “DNA Extraction and Identification of the Isolates,” and sequenced in the Ion Torrent Personal Genome Machine (Thermo Fisher Scientific) using chip 318 v.2 according to the manufacturer’s protocol. The quality of the reads was visualized using FastQC^2^. The reads were trimmed, discarding bases with Phred values below 20, and filtered, discarding reads with less than 100 nucleotides. The reads were assembled in contigs using the software MIRA 4 (Chevreux et al., 2004). Redundant contigs were removed using the SeqMan Pro tool of the Lasergene software (DNASTAR). The sequenced genomes were submitted to the GenBank database under the accession numbers PKCA01000000 (*E. coli* APC43A) and PYSX01000000 (*A. baumannii* APC25).

The contigs were ordered in scaffolds with MAUVE (Darling et al., 2004). Automatic genome annotation was performed in RAST (Rapid Annotation using System Technology) (Aziz et al., 2008). The RAST SEED subsystems (Overbeek et al., 2014), CARD (Comprehensive Antibiotic Resistance Database) (McArthur et al., 2013) and Resfinder v.2.1 (Zankari et al., 2012) were used to search for resistance genes in the sequenced genomes.

An *in silico* analysis of Plasmid Multilocus Sequence Typing (MLST) was performed using the web tool pMLST v.1.8 (Larsen et al., 2012) available at the site of the Center for Genomic Epidemiology^3^. PlasmidFinder v.1.3 (Carattoli et al., 2014) was used for detection of plasmid sequences, PathogenFinder v.1.1 (Cosentino et al., 2013) was used to determine the strains’ pathogenicity level, SerotypeFinder v.1.1 (Joensen et al., 2015) was used for serotyping, and VirulenceFinder v.1.5 (Joensen et al., 2014) was used to detect virulence determinants.

Pathogenicity Islands (PAIs) and Resistance Islands (RIs) were predicted using the software GIPSy v.1.1.2 (Soares et al., 2016). *E. coli* K-12 substr. MG1655 (NC_000913.3) and *Acinetobacter calcoaceticus* CA16 (NZ_CP020000.1) were used as reference strains. The nucleotide sequence of each PAI and RI were recovered using the genome browser Artemis v.14.0.0 (Rutherford et al., 2000). In order to determine the location of PAIs and RIs, we designed a circular map using BLASTn in the software BRIG (Blast Ring Image Generator) (Alikhan et al., 2011).

A phylogenomic approach was used to determine the isolates species affiliation. Genomes used for comparison were obtained from GenBank. Four phylogenetic markers: 16S rRNA, *rpoB*, *gyrB*, and *dnaJ* were used to calculate a distance matrix based on a BLASTn comparison all-against-all in the software Gegenees v.2.2.1 (Ågren et al., 2012).

## Results and Discussion

### Water Quality and Characterization of Cultivable Antibiotic-Resistant Bacteria

The majority of physical, chemical and microbiological parameters were within the limits established by the Brazilian law for freshwater environments intended for human consumption after appropriate treatment (Supplementary Table S1). However, BOD in sampling points 1, 4, 5, and 6 was above the recommended values. Additionally, dissolved oxygen (DO) concentration was below the limit in all sampling points analyzed. These two results suggest high oxygen consumption by the microbial community in Lake Água Preta during the sampling period.

For this study, sampling was performed only in October, and seasonal variation was not assessed. The temperature in this geographic region, immediately below the equator, is high throughout the year, though there are significant differences in terms of rainfall. The decision to sample in the dry season (July to November) was due to logistics issues related to lake access. However, in future studies it would be interesting to evaluate seasonal factors that may affect water quality and antibiotic resistance in Lake Água Preta.

Thirty-three isolates were obtained in this study (Table 1). Isolates affiliated mostly to genus *Escherichia* (7 isolates), followed by genera *Acinetobacter*, *Enterobacter*, and *Klebsiella* (5 isolates each), *Pseudomonas* (4 isolates), *Shigella* (3 isolates), and *Chromobacterium*, *Citrobacter*, *Leclercia* and *Phytobacter* (1 isolate each).

**Table 1:** Characteristics of isolates obtained from Lake Água Preta.

Isolate	16S rRNA gene Affiliation	Resistance (and intermediate) phenotype	Resistance genotype	Integrase genes
APC43A	*Escherichia coli* NBRC 102203 (99%)	AML, AMC, AMP, CEF, CAZ, CTX, FEP, ATM, CIP, NAL (KAN)^b^	*bla*_CTX–M–15_^a^	–
APC25	*Acinetobacter baumannii* DSM 30007 (97%)	AML, AMC, AMP, CEF, CAZ, CTX, ATM, CIP, NAL (KAN)	–^a^	–
APC4	*Citrobacter werkmanii* CDC 0876-58 (99%)	AML, AMC, AMP, CEF, CAZ, CTX, ATM, CIP, NAL, SXT, TET (KAN)	–	–
APC6	*Enterobacter sp.* A2 (99%)	AML, AMC, AMP, CEF, CAZ, ATM, CTX, CIP, GEN, KAN, NAL, TET	*bla*_TEM-_*bla*_SHV_	–
APC11	*Pseudomonas putida* F1 (99%)	AML, AMC, AMP, CEF, CAZ, CTX, ATM, CHL, NAL, SXT	*bla*_CTX–M_	–
APC13	*Acinetobacter baumannii* DSM 30007 (97%)	AML, AMP, CEF, CAZ, FEP, ATM (CTX, NAL)	–	–
APC14	*Pseudomonas mosselii* CFML 90-83 (99%)	AML, AMC, AMP, CEF, CAZ, CTX, FEP, ATM, TET (NAL)	–	–
APC15	*Chromobacterium haemolyticum* MDA0585 (99%)	AML, AMC, AMP, CEF, CAZ, CTX, FEP, IPM, ATM, KAN, NAL	–	–
APC19	*Escherichia coli* O157:H7 Sakai (99%)	AML, AMC, AMP, CEF, CAZ, CTX, FEP, ATM, CIP, GEN, KAN, NAL, SXT, TET	*bla*_CTX–M_	*intI*2
APC20	*Pseudomonas mosselii* CFML 90-83 (99%)	AML, AMC, AMP, CEF, CAZ, CTX, FEP, ATM (NAL)	–	–
APC22	*Shigella sonnei* Ss046 (99%)	AML, AMP, CEF, CTX, FEP, ATM, CIP, GEN, KAN, NAL, SXT (AMC)	*bla*_CTX–M–2_	*intI*2
APC24B	*Escherichia fergusonii* ATCC 35469 (99%)	AML, AMC, AMP, CEF, CAZ, CTX, FEP, ATM, CIP, KAN, NAL, SXT, TET	*bla*_CTX–M_	*IntI*1
APC28	*Klebsiella pneumoniae* DSM 30104 (96%)	AML, AMC, AMP, CEF, CAZ, CTX, FEP, ATM, CIP, KAN, NAL, SXT, TET (C)	*bla*_TEM-_ *bla*_SHV_*-bla*_CTX–M–15_	*intI*1
APC32	*Escherichia fergusonii* ATCC 35469 (97%)	AML, AMP, CEF, CAZ, CTX, FEP, ATM, SXT (AMC, CIP, KAN)	*bla*_TEM_*-bla*_CTX–M–15_	–
APC33	*Shigella sonnei* Ss046 (99%)	AML, AMC, AMP, CEF, CAZ, CTX, FEP, ATM, CIP, NAL	*bla*_CTX–M–15_	–
APC34	*Escherichia coli* NBRC 102203 (98%)	AML, AMC, AMP, CEF, CAZ, CTX, FEP, ATM, CIP, KAN, NAL, SXT, TET	*bla*_CTX–M_	*IntI*2
APC38	*Escherichia fergusonii* ATCC 35469 (98%)	AML, AMC, AMP, CEF, CTX, FEP, ATM, CIP, GEN, CHL, KAN, NAL, SXT, TET	*bla*_CTX–M_	*IntI*2
APC39	*Acinetobacter nosocomialis* RUH2376 (99%)	AML, AMC, AMP, CEF, CAZ, CTX, ATM, CIP, KAN, NAL, SXT, TET	–	–
APC40A	*Escherichia fergusonii* ATCC 35469 (99%)	AML, AMC, AMP, CEF, CAZ, CTX, FEP, ATM, CIP, GEN, CHL, NAL, SXT (KAN)	*bla*_CTX–M–14_	*intI*1-*intI*2
APC42	*Acinetobacter baumanii* DSM 30007 (99%)	AML, AMC, AMP, CEF, CAZ, CTX, FEP, ATM, CIP, KAN, NAL, SXT, TET (CHL)		–
APC43B	*Shigella sonnei* Ss046 (99%)	AML, AMC, AMP, CEF, CAZ, CTX, FEP, ATM, CIP, GEN, KAN, NAL, SXT, TET (CHL)	*bla*_CTX–M–15_	–
API2	*Klebsiella pneumoniae* R-70 (99%)	AML, ATM, C, KAN, SXT, TET (AMP, CIP)	–	*IntI*1
API3	*Enterobacter asburiae* JCM6051 (94%)	AML, AMC, AMP, CEF, CAZ, ATM, CHL, KAN, NAL, TET	–	–
API4	*Klebsiella pneumoniae* 07A044 (99%)	AML, AMC, AMP, CEF, ATM, SXT, TET (CTX, NAL)	*bla*_SHV_	–
API6	*Leclercia adecarboxylata* CIP 82.92 (99%)	AML, AMC, AMP, CEF, ATM, CHL, KAN, NAL, TET (CTX)	–	–
API7	*Enterobacter cloacae* LMG 2683 (99%)	AML, AMC, AMP, CEF (CTX, ATM)	–	–
API10	*Enterobacter tabaci* YIM Hb-3 (99%)	AML, AMC, AMP, CEF, ATM, CHL, KAN, NAL (SXT, TET)	–	–
API12	*Pseudomonas otitidis* MCC10330 (99%)	AML, AMC, AMP, CEF, ATM, KAN, TET (NAL)	–	–
API16	*Enterobacter tabaci* YIM Hb-3 (100%)	AML, AMC, AMP, CEF, ATM, GEN, CHL, NAL, SXT, TET (CTX)	–	–
API20	*Acinetobacter seifertii* LUH 1472 (99%)	AML, AMC, AMP, CEF, ATM, CIP, KAN, TET (CAZ, CTX, GEN, CHL)	–	–
API24	*Phytobacter diazotrophicus* Ls8 (98%)	AMP, CAZ, CTX, ATM, KAN, NAL (AML, FEP, TET)	*bla*_TEM_	–
API29	*Klebsiella pneumoniae* DSM 30104 (99%)	AML, AMP, CEF	*bla*_TEM-_ *bla*_SHV_	–
API34	*Klebsiella pneumoniae* DSM 30104 (98%)	AML, AMP, CEF, CTX, FEP, ATM, CIP, GEN, CHL, NAL, SXT, TET	–	–

*The BLASTn identity result for each isolate is presented within parentheses after the 16S rRNA affiliation. The abbreviation of antibiotics is as follows: amoxicillin (AML); amoxicillin + clavulanic acid (AMC); ampicillin (AMP); cephalotin (CEF); ceftazidime (CAZ); cefotaxime (CTX); aztreonam (ATM); cefepime (FEP); imipenem (IPM); kanamycin (KAN); gentamicin (GEN); nalidixic acid (NAL); ciprofloxacin (CIP); chloramphenicol (CHL); tetracycline (TET); sulfamethoxazole + trimethoprim (SXT). ^a^Isolates selected for whole genome analysis. The complete analysis of its resistance genotype is presented in main text and in Table 2. ^b^Parentheses indicate intermediate susceptibility to the antibiotic.*

Most isolates were classified as multi-drug resistant (29/33–88%), meaning resistant to at least three classes of antibiotics. All isolates showed resistance to penicillins such as amoxicillin, ampicillin or both (Table 1), and 79% were also resistant when the penicillin (amoxicillin) was combined with a beta-lactamase inhibitor (clavulanic acid). Twenty-one of the 33 isolates showed resistance to cefotaxime (63.6%) and six showed intermediate resistance (18.2%). Resistance to carbapenems was detected only in the *Chromobacterium* isolate (Table 1). This genus has been commonly isolated from aquatic ecosystems and presents intrinsic resistance to these last-resort antibiotics (Lima-Bittencourt et al., 2011). The importance of *Chromobacterium* as progenitor of KPC carbapenemases has been recently discussed (Gudeta et al., 2016). For non-beta-lactam antibiotics, high levels of resistance or intermediate resistance were observed against aminoglycosides (76% of resistant isolates), tetracycline (64%), ciprofloxacin (58%) and the combination trimethoprim/sulfamethoxazole (55%). These results are in accordance with previous studies, which reported high levels of multi-drug resistance among strains resistant to third generation cephalosporins (Tacão et al., 2014). The presence of multi-drug resistant bacteria in natural aquatic systems may result from several anthropogenic pressures (Taylor et al., 2011; Tacão et al., 2012). The values of BOD and DO within Lake Água Preta are consistent with an impacted environment. An important cause may be the disposal of untreated sewage, resulting from an increasing number of illegal houses constructed along the margins. As in other geographic locations (e.g., Alves et al., 2014), wild life may also contribute to antibiotic resistance spread in this region. Finally, the presence of sub-lethal concentrations of antibiotics in aquatic systems has been reported to select for antibiotic resistant bacteria. In Brazil, until recently, antibiotics were among the most consumed medical drugs, and sold without medical prescription (Mattos et al., 2017). This situation might have contributed to the contamination of aquatic systems. These systems have been reported to act as reservoirs and to promote the transfer of antibiotic resistance genes among bacteria, thus contributing to multi-drug resistance spread.

The most frequently detected beta-lactamase gene was *bla*_CTX–M_ (*n* = 12 positive isolates), followed by *bla*_TEM_ (*n* = 5) and *bla*_SHV_ (*n* = 4) (Table 1). As in our study, CTX-M is the most frequently reported ESBL worldwide (Tacão et al., 2012; Bevan et al., 2017). Carbapenemase genes *bla*_IMP_, *bla*_V IM_, and *bla*_KPC_ were not detected among the isolates. Of the 22 isolates resistant to third generation cephalosporins, the gene *bla*_CTX–M_ was not detected in 10. These isolates affiliated to the genera *Acinetobacter* (*n* = 3), *Pseudomonas* (*n* = 2), *Citrobacter* (*n* = 1), *Enterobacter n* = 1), *Phytobacter* (*n* = 1), *Chromobacterium* (*n* = 1) and *Klebsiella* (*n* = 1). The *bla*_SHV_ is known to be intrinsic to *Klebsiella pneumoniae* (Babini and Livermore, 2000). Although we have used two sets of primers targeting this gene, under the conditions tested it was not detected in two of the isolates that affiliated with this species, including isolate API34 which showed resistance to cefotaxime. This result may be related to primer-template mismatches or to the affiliation of these isolates to a different *Klebsiella* species. Resistance to cefotaxime in *Klebsiella* spp. may be related with overproduction of other chromosomal beta-lactamases (e.g., *bla*_OXY_, *bla*_LEN_, *bla*_OKP_) due to mutations in the gene promoter regions (Hæggman et al., 2004). Overexpression of chromosomal beta-lactamases may also be the mechanism responsible for resistance to third-generation cephalosporins in isolates affiliated to other bacterial genera such as *Enterobacter*, *Citrobacter*, *Chromobacterium*, and *Pseudomonas* (intrinsic *bla*_AmpC_; Jacoby, 2009), or *Acinetobacter* (e.g., *bla*_ADC_ genes; Zhong et al., 2008). The *bla*_CTX–M–15_ gene was found in 5 isolates (affiliated with genera *Klebsiella*, *Escherichia* and *Shigella*), the *bla*_CTX–M–14_ gene was found in 1 isolate (affiliated with *Escherichia*), and the *bla*_CTX–M–2_ gene was detected in only 1 isolate (affiliated with *Shigella*). These variants have previously been reported in Brazil in both clinics and environmental settings (Dropa et al., 2016; Rocha et al., 2016; Nascimento et al., 2017; Sellera et al., 2017). For the remaining *bla*_CTX–M_-positive isolates, it was only possible to sequence a portion of the gene, insufficient to accurately determine its variant. For these isolates, PCR products were not obtained with the primers used to characterize the genomic context of *bla*_CTX–M_. IS*Ecp1* was found in the upstream region of all *bla*_CTX–M–15_ and *bla*_CTX–M–14_ genes. Downstream, all *bla*_CTX–M–15_ genes presented Orf477 and *bla*_CTX–M–14_ presented the insertion sequence IS*903*. The same contexts were previously reported for these genes in clinical and environmental isolates worldwide (Eckert et al., 2006; Tacão et al., 2012). Particularly, the association of IS*Ecp1* element with ESBL genes seems to be one of the reasons for the successful spread of these genes, being a major concern in clinical settings (Tian et al., 2011). The genetic context of *bla*_CTX–M–2_ carried by *Shigella* sp. APC22 was identical to that previously described (Eckert et al., 2006): an upstream region with a *sul1* gene (encoding resistance to sulfonamides) followed by an IS*CR1* element; and downstream an open reading frame designated Orf3, followed by *qacEdelta1* (encoding for a quaternary ammonium compound resistance protein) and a *sul1* gene. These CR-like elements are usually associated to complex class 1 integrons, usually identified between duplications of 3’conserved sequence (CS) regions, along with antibiotic resistance genes like *bla*_CTX–M–2_ (Toleman et al., 2006).

Conjugations assays were performed for nine out of twelve *bla*_CTX–M_-positive isolates. Three isolates were able to grow on rifampicin and were excluded from these experiments. Under the used conjugation conditions, 4 out of 9 donor strains generated transconjugants carrying *bla*_CTX–M_. In contrast with the recipient strain *E. coli* CV601, all transconjugants showed MIC for cefotaxime from 32 to >256 μg/mL, while for ceftazidime MICs varied from 2 to 8 μg/mL (Supplementary Table S2). Overall, the association of *bla*_CTX–M_ genes to conjugative plasmids in these isolates was confirmed indicating that their mobilization to different hosts may be facilitated.

Previous studies highlighted the important contribution of integrons to multi-drug resistance profiles among ESBL-producers (Tacão et al., 2014). In this study, the integrase genes *intI*1 and *intI*2 were detected in 4 and 5 isolates, respectively (Table 1). All but one of these isolates were positive for the *bla*_CTX–M_ gene.

### Genomic Analysis of Two Multi-Drug Resistant Isolates

To obtain an in-depth characterization of the resistome of selected isolates, as well as insights into their mobilome and virulence potential, two isolates (i.e., *E. coli* APC43A and *Acinetobacter baumannii* APC25) were selected for whole genome sequencing. These isolates were randomly selected among isolates that: (1) belong to bacterial groups of public health concern, (2) presented multi-drug resistance profiles.

Identification at species level was confirmed using a phylogenomic approach as described in Material and Methods. Both strains were resistant to all beta-lactams except to imipenem (APC43A) or to imipenem and cefepime (APC25). Additionally, strains showed resistance to ciprofloxacin, nalidixic acid, and an intermediate susceptibility to kanamycin. Summary of both strains genomic features is presented in Table 2.

**Table 2:** Major genomic features of two isolates from Lake Água Preta and resistance genes annotated by CARD and/or ResFinder.

*E. coli* APC43A	%GC	CDS	contigs	N50	RIs	PAIs	Size (bp)	MLST	Serotype	Plasmids
	50.5	5923	195	3283348	5	25	5,035,455	ST471	O154:H18	IncX4 e IncFIA

**Resistance Genes**	**ARO category**							**Genes**	**Contig localization**	

	Antibiotic inactivation enzyme; beta-lactam resistance proteins							*bla*_CTX–M–15_	22_18911	
								intrinsic *bla*_AmpC_		
	Efflux pump conferring antibiotic resistance							*acrE*	15_8461	
								*emrB*	10_26638	
								*mdtB*	5_4183	
								*mdtL*	8_54031	
								*msbA*	11_125192	
								*tolC*	7_57761	
	Efflux pump conferring antibiotic resistance; antibiotic resistance gene cluster, cassette, or operon							*mdtE*	37_5848	
	Efflux pump conferring antibiotic resistance; gene modulating antibiotic efflux							*acrS*	15_10016	
								*cpxR*	19_63902	
								*emrR*	10_24793	
								*H-NS*	20_22613	

***A. baumanii* APC25**	**%GC**	**CDS**	**contigs**	**N50**	**RIs**	**PAIs**	**Size (bp)**	**MLST**	**Serotype**	**Plasmids**

	39.0	5063	121	244684	10	11	4,860,843	–^a^	–^a^	–^b^

**Resistance Genes**	**ARO category**							**Genes**	**Contig localization**	

	Efflux pump complex or subunit conferring antibiotic resistance							*adeK*	11_73107	
	Antibiotic inactivation enzyme; beta-lactam resistance protein							*bla*_OXA–208_	23_160179	
								*bla*_ADC–like_	6_85724	

*^a^pMLST and SeroTypeFinder do not have support for this taxon. ^b^PlasmidFinder did not detected any plasmid sequence.*

#### *Escherichia coli* APC43A Genomic Analysis

**FIGURE 2:**
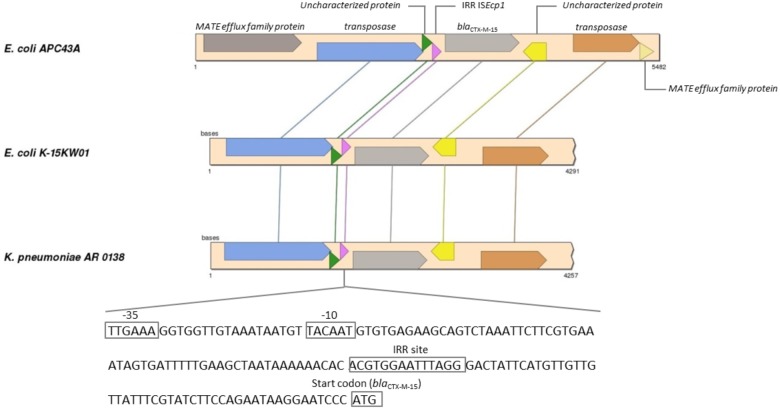
Synteny analysis of IS*Ecp1* element carrying *bla*_CTX–M–15_ gene in *Escherichia coli* APC43A. Conserved genes are connected by lines. The pink arrow represents the IS*Ecp1* element that is described in detail at the bottom of the figure. *E. coli* APC43A contains two CDSs identified as MATE efflux family proteins flanking the IS element. These CDSs are complementary and probably are part of the same gene that was fragmented during the insertion of the IS element.

For *E. coli* APC43A the RAST server classified 162 CDSs in the subsystem of Virulence, Disease and Defense (3.2% of total genes) (Supplementary Table S3). Among them, 122 were genes related to antibiotic resistance and toxic compounds. Two beta-lactamase genes were predicted in the genome. As described above, *bla*_CTX–M–15_ gene was located between IS*Ecp1* and orf477. Genomic analysis revealed that a transposase gene followed orf477 and that two fragments of a truncated gene encoding a MATE efflux family protein flanked this entire region (Figure 2). This region showed identity values higher than 99% and coverage higher than 93% with the genomes of *K. pneumoniae* AR 0138 (CP021757.1) and *E. coli* K-15KW01 (CP016358.1) (Zurfluh et al., 2016). In *E. coli* K-15KW01 the *bla*_CTX–M–15_ gene was embedded at the right-hand extremity of an IS*Ecp1* element (Figure 2). In our strain APC43A, the inverted repeat sequence (IRR) (ACGTGGAATTTAGG), and the −35 (TTGAAA) and −10 (TACAAT) sites of the IS*Ecp1* element were conserved 48 base pairs upstream of the ATG start codon of *bla*_CTX–M–15_ (Figure 2). The annotation of the other identified beta-lactamase gene was evaluated by comparing its nucleotide sequence to the Uniprot database through BLASTn. The gene showed high identity with an intrinsic AmpC beta-lactamase encoding gene (above 99%), emphasizing its correct annotation. Mutations previously related to enzyme overexpression (Jacoby, 2009) were not detected in the *bla*_AmpC_ gene promoter. Besides beta-lactamase genes, genes encoding resistance to other classes of antibiotics were detected in the genome of strain APC43A, mostly related with efflux pumps (Table 2).

Sequences representing two plasmids, assigned to the incompatibility groups IncX4 and IncFIA, were detected in the genome of *E. coli* APC43A (Table 2). The contig corresponding to replicon IncX4 has a size of 30,306 bp, which is very similar to the size of *E. coli* IncX4 plasmids in the GenBank database (e.g., accession number JX981514.1). This plasmid was detected in the porcine enterotoxigenic strain *E. coli* UMNF18 carrying genes for type II secretion system (Shepard et al., 2012). IncFIA is a fertility plasmid of *E. coli* and part of this plasmid was detected in a 9,933 bp contig. No resistance genes were found within plasmids.

PathogenFinder analysis showed that *E. coli* APC43A is a human pathogen and the SerotypeFinder tool classified this strain in the O154:H18 serotype. Six virulence factors (*gad*, *lpfA*, *ltcA*, *astA*, *cba* e *cma*) normally found in pathogenic *E. coli* were detected in the genome of *E. coli* APC43A. These virulence genes are involved in host-pathogen interaction during gastrointestinal infections caused by ingestion of contaminated food or water (Joensen et al., 2014). The strain was assigned to ST471, a high-risk clone previously reported in clinical settings and commonly associated with ESBL genes and genes encoding carbapenemases (Kapmaz et al., 2016; Yi et al., 2017). In Brazil, this sequence type was described in clinical isolates from Rio de Janeiro (Peirano et al., 2011).

#### *Acinetobacter baumannii* APC25 Genomic Analysis

High levels of intrinsic resistance to a number of antibiotics have been reported for *A. baumanii*, seriously compromising the treatment of patients infected with this pathogen. Intrinsic resistance mechanisms in members of this species include the production of chromosomal beta-lactamases and aminoglycoside-modifying enzymes, expression of efflux pumps and permeability defects. Nevertheless, *A. baumanii* is also known for its ability to acquire genes encoding resistance determinants.

For the genome of *A. baumannii* APC25 the RAST server classified 109 CDSs in the subsystem of Virulence, Disease and Defense (2.6% of total genes) (Supplementary Table S4). Eighty-three of these 109 CDSs are related to resistance to antibiotics and toxic compounds. The beta-lactamase genes *bla*_OXA–208_ and *bla*_ADC–like_ (98% similar to *bla*_ADC–25_) were detected (Table 2). Both genes were previously reported as intrinsic genetic determinants in the chromosome of *A. baumannii* (Zhao and Hu, 2012). *bla*_OXA–208_ encodes an OXA-51-like chromosomally encoded beta-lactamase (Evans and Amyes, 2014). Clinically relevant oxacillinases have been reported in clinical isolates from sixteen states in Brazil, mostly OXA-23 and OXA-143 (Medeiros and Lincopan, 2013). The *bla*_ADC–25_ encodes a cephalosporinase recently described to confer resistance to second and third generation cephalosporins (Zhong et al., 2008; Lee et al., 2012), a result that is in line with the antibiotic susceptibility profile of strain APC25.

Plasmids were not detected in *A. baumannii* APC25 and the isolate was not predicted as a human pathogen by the PathogenFinder tool (Supplementary Table S5). MLST sequences were uploaded to the *Acinetobacter*-MLST Pasteur database and since an unreported allele combination was observed, a new sequence type (ST1278) was assigned.

#### Resistance to Metals and Genomic Islands Prediction

Operons related to resistance to metals were determined in the sequenced strains. *E. coli* APC43A possesses incomplete mercury resistance operons (Supplementary Figure S1). In addition, the two-component system *cusR*-*cusS* and the efflux pump *cusCFBA*, described as responsible for copper and silver resistance in other strains of *E. coli* (Gudipaty and McEvoy, 2014), were annotated in the genome. In *A. baumannii* APC25, the zinc, cadmium, and cobalt resistance may be mediated by the operon *czcABC*, which was found duplicated in its genome (Supplementary Figure S1). Both genomes showed operons for resistance to arsenic. *A. baumannii* APC25 has an operon composed by an arsenical resistance-3 (ACR3) family protein, while *E. coli* APC43A has an *arsRBC* type operon (Supplementary Figure S1). Several studies have showed that some pollutants such as metals could co-select for antibiotic resistance (Wright et al., 2008; Rosewarne et al., 2010; Henriques et al., 2016). However, the level of aluminum, manganese, nickel, cadmium, copper and zinc in Lake Água Preta was in accordance to the standard values for mesotrophic lakes (Supplementary Table S1).

**FIGURE 3:**
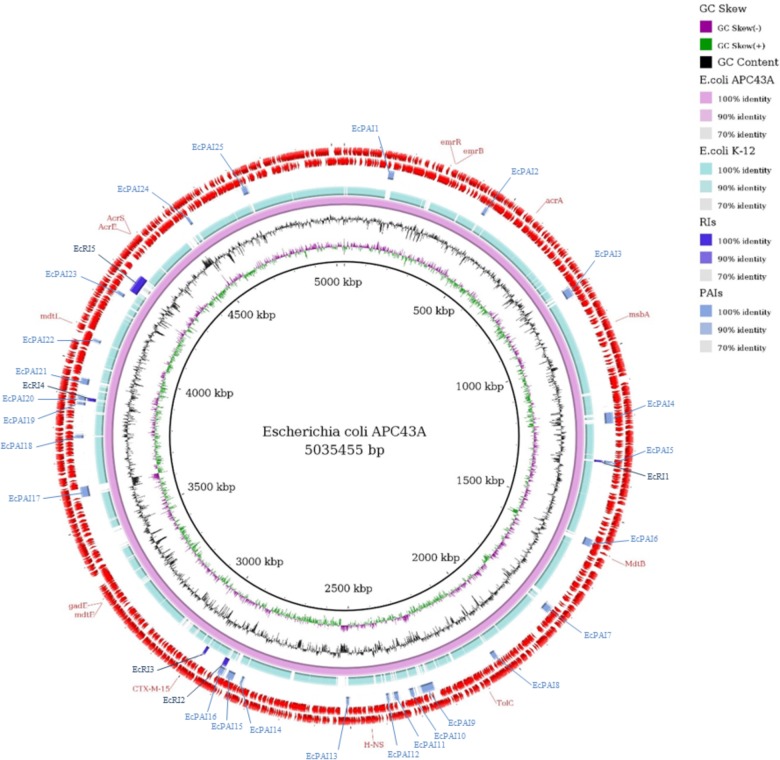
Comparative genomic ring designed in Gegenees program. The innermost ring to the outermost is presented in this figure, as follows: GC skew and the GC content of *E. coli* APC43A; the genome sequence of *E. coli* APC43A and *E. coli* K-12; the Resistance Islands (RIs) detected by GIPSy; the Pathogenicity Islands (PAIs) detected by GIPSy; CDSs identified in the genome of *E. coli* APC43A. The location of the resistance genes detected by CARD and ResFinder are shown and identified by their respective names in red.

**FIGURE 4:**
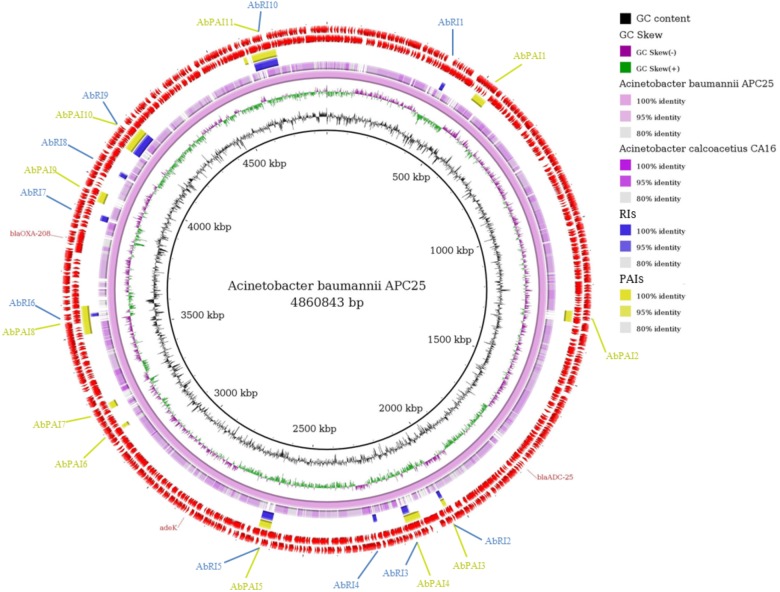
Comparative genomic ring designed in Gegenees program. The innermost ring to the outermost is presented in this figure, as follows: GC skew and the GC content of *Acinetobacter baumannii* APC25; the genome sequence of *A. baumannii* APC25 and *A. calcoacetius* CA16; the Resistance Islands (RIs) detected by GIPSy; the Pathogenicity Islands (PAIs) detected by GIPSy; CDSs identified in the genome of *A. baumannii* APC25. The location of the resistance genes detected by CARD and ResFinder are shown and identified by their respective names in red.

Twenty-five PAIs and five RIs were identified in the genome of *E. coli* APC43A (Figure 3). The location of the islands is shown in the comparative ring of Figure 4. It is worth noting that these islands are almost completely absent in the genome of the non-pathogenic *E. coli* K-12 (Figure 3). Interestingly, among the detected resistance genes only the gene *mdtB* was within a GEI (EcPAI16), suggesting that these resistance islands may encode resistance to other classes of compounds. In some cases, the program identified PAIs and RIs in the same genome region, e.g., EcPAI5 and EcRI1, which means that these regions may encode both resistance and virulence factors.

The genome of *A. baumannii* APC25 has 11 PAIs and 10 RIs (Figure 4). The low number of PAIs is in accordance with the prediction of PathogenFinder that classified the isolate as a non-pathogenic strain. The majority of PAIs and RIs were found in the same location of the genome similar to that observed for *E. coli* (Figure 4). No resistance genes predicted by CARD or ResFinder were located within GEI.

## Conclusion

Lake Água Preta is an Amazonian mesotrophic lake located near a densely populated area that presented physical, chemical and microbiological parameters in accordance to the Brazilian environmental laws, with some exceptions. The majority of bacterial strains (29 out of 31; 88%) isolated from the lake, in media supplemented with cefotaxime, were multi-drug resistant, classified in the Enterobacteriaceae family, and carried ESBL genes, primarily *bla*_CTX–M_. In some cases the transfer potential of these genes were confirmed in conjugation assays. These results suggest a high dissemination of ESBL genes in Gram-negative bacteria of Lake Água Preta, which although not presenting characteristics of a highly impacted environment, contains multi-drug resistant pathogenic strains such as *E. coli* APC43A (ST471).

## Author Contributions

AS, IH, AF, and RB conceived and designed the experiments. DF, SA, JA, and MT performed the experiments. DF, RR, and RB were involved in genome analysis. DF, SA, MT, RB, and IH prepared the manuscript.

## Conflict of Interest Statement

The authors declare that the research was conducted in the absence of any commercial or financial relationships that could be construed as a potential conflict of interest.

## Footnotes

1 http://www.ncbi.nlm.nih.gov/

2 http://www.bioinformatics.babraham.ac.uk/projects/fastqc/

3 http://www.genomicepidemiology.org/

## References

[B1] Ågren J. Sundström A. Håfström T. Segerman B. (2012). Gegenees: fragmented alignment of multiple genomes for determining phylogenomic distances and genetic signatures unique for specified target groups. *PLoS One* 7:e39107. 10.1371/journal.pone.0039107 22723939 PMC3377601

[B2] Alikhan N. F. Petty N. K. Bem Zakour N. L. Beatson S. A. (2011). BLAST Ring Image Generator (BRIG): simple prokaryote genome comparisons. *BMC Genomics* 12:402. 10.1186/1471-2164-12-402 21824423 PMC3163573

[B3] Alves M. S. Pereira A. Araujo S. M. Castro B. B. Correia A. C. M. Henriques I. (2014). Seawater is a reservoir of multi-resistant *Escherichia coli*, including strains hosting plasmid-mediated quinolones resistance and extended-spectrum beta-lactamases genes. *Front. Microbiol.* 5:426. 10.3389/fmicb.2014.00426 25191308 PMC4138442

[B4] Ambler R. P. (1980). Structure of β-lactamases. *Philos. Trans. Royal Soc. London B Biol. Sci.* 289 321–331. 10.1098/rstb.1980.0049 6109327

[B5] Aziz R. K. Bartels D. Best A. A. DeJongh M. Disz T. Edwards R. A. (2008). The RAST Server: rapid annotations using subsystems technology. *BMC Genomics* 9:75. 10.1186/1471-2164-9-75 18261238 PMC2265698

[B6] Babini G. S. Livermore D. (2000). Are SHV β-Lactamases universal in *Klebsiella pneumoniae*? *Antimicrob. Agents Chemother.* 44:2230. 10.1128/aac.44.8.2230-2230.2000 PMC90049 11023444

[B7] Baquero F. Martinez J. L. Cantón R. (2008). Antibiotics and antibiotic resistance in water environments. *Curr. Opin. Biotechnol.* 19 260–265. 10.1016/j.copbio.2008.05.006 18534838

[B8] Bauer A. W. Kirby W. M. Sherris J. C. Turck M. (1966). Antibiotic susceptibility testing by a standardized single disk method. *Tech. Bull. Regist. Med. Technol.* 36 49–52. 5908210

[B9] Bevan E. R. Jones A. M. Hawkey P. M. (2017). Global epidemiology of CTX-M beta-lactamases: temporal and geographical shifts in genotype. *J. Antimicrob. Chemother.* 72 2145–2155. 10.1093/jac/dkx146 28541467

[B10] Carattoli A. Zankari E. García-Fernández A. Voldby Larsen M. Lund O. Villa L. (2014). In silico detection and typing of plasmids using Plasmid Finder and plasmid multilocus sequence typing. *Antimicrob. Agents Chemother.* 58 3895–3903. 10.1128/AAC.02412-14 24777092 PMC4068535

[B11] Chevreux B. Pfisterer T. Drescher B. Driesel A. J. Muller W. E. Wetter T. (2004). Using the miraEST assembler for reliable and automated mRNA transcript assembly and SNP detection in sequenced ESTs. *Genome Res.* 14 1147–1159. 15140833 10.1101/gr.1917404 PMC419793

[B12] CLSI (2017). *Performance Standard for Antimicrobial Susceptibility Testing - Document Approved Standard M100-S27.* Wayne, PA: CLSI.

[B13] CONAMA (2005). *Ministério do Meio Ambiente Conselho Nacional do Meio Ambiente. Resolução CONAMA n° 357/2005.* Brasília: Diário Oficial da República Federativa do Brasil.

[B14] Cosentino S. Voldby Larsen M. Møller Aarestrup F. Lund O. (2013). PathogenFinder - distinguishing friend from foe using bacterial whole genome sequence data. *PLoS One* 8:e77302. 10.1371/journal.pone.0077302 24204795 PMC3810466

[B15] Dallenne C. Da Costa A. Decré D. Favier C. Arlet G. (2010). Development of a set of multiplex PCR assays for the detection of genes encoding important beta-lactamases in *Enterobacteriaceae*. *J. Antimicrob. Chemother.* 65 490–495. 10.1093/jac/dkp498 20071363

[B16] Darling A. C. E. Mau B. Blattner F. R. Perna N. T. (2004). Mauve: multiple alignment of conserved genomic sequence with rearrangements. *Genome Res.* 14 1394–1403. 15231754 10.1101/gr.2289704 PMC442156

[B17] de Oliveira D. V. Nunes L. S. Barth A. L. Van Der Sand S. T. (2017). Genetic background of beta-lactamases in *Enterobacteriaceae* isolates from environmental samples. *Microbial Ecol.* 74 599–607. 10.1007/s00248-017-0970-6 28378066

[B18] Dropa M. Lincopan N. Balsalobre L. C. Oliveira D. E. Moura R. A. Fernandes M. R. (2016). Genetic background of novel sequence types of CTX-M-8- and CTX-M-15-producing *Escherichia coli* and *Klebsiella pneumoniae* from public wastewater treatment plants in São Paulo, Brazil. *Environ. Sci. Pollut. Res. Int.* 23 4953–4958. 10.1007/s11356-016-6079-5 26782324

[B19] ECDC (2017). *European Centre for Disease Prevention and Control. Antimicrobial resistance surveillance in Europe 2016. Annual Report of the European Antimicrobial Resistance Surveillance Network (EARS-Net).* Stockholm: ECDC.

[B20] Eckert C. Gautier V. Arlet G. (2006). DNA sequence analysis of the genetic environment of various blaCTX-M genes. *J. Antimicrob. Chemother.* 57 14–23. 16291869 10.1093/jac/dki398

[B21] Evans B. A. Amyes S. G. B. (2014). OXA beta-Lactamases. *Clin. Microbiol. Rev.* 27 241–263. 10.1128/CMR.00117-13 24696435 PMC3993105

[B22] Gudeta D. D. Bortolaia V. Jayol A. Poirel L. Nordmann P. Guardabassi L. (2016). *Chromobacterium* spp. harbour Ambler class A beta-lactamases showing high identity with KPC. *J. Antimicrob. Chemother.* 71 1493–1496. 10.1093/jac/dkw020 26892778

[B23] Gudipaty S. A. McEvoy M. M. (2014). The histidine kinase CusS senses silver ions through direct binding by its sensor domain. *Biochim. Biophys. Acta* 1844 1656–1661. 10.1016/j.bbapap.2014.06.001 24948475 PMC4116807

[B24] Hall T. (1999). Bioedit: a user-friendly biological sequence alignment editor and analysis program for Windows 95/96/NT. *Nucleic Acids Ser.* 41 95–98.

[B25] Henriques I. Tacão M. Leite L. Fidalgo C. Araújo S. Oliveira C. (2016). Co-selection of antibiotic and metal(loid) resistance in gram-negative epiphytic bacteria from contaminated salt marshes. *Mar. Pollut. Bull.* 109 427–434. 10.1016/j.marpolbul.2016.05.031 27210560

[B26] Hæggman S. Löfdahl S. Paauw A. Verhoef J. Brisse S. (2004). Diversity and evolution of the class A chromosomal beta-lactamase gene in *Klebsiella pneumoniae*. *Antimicrob. Agents Chemother.* 48 2400–2408. 10.1128/AAC.48.7.2400-2408.2004 15215087 PMC434173

[B27] Jacoby G. A. (2009). AmpC -Lactamases. *Clin. Microbiol. Rev.* 22 161–182. 10.1128/CMR.00036-08 19136439 PMC2620637

[B28] Jiao Y. N. Chen H. Gao R. X. Zhu Y. G. Rensing C. (2017). Organic compounds stimulate horizontal transfer of antibiotic resistance genes in mixed wastewater treatment systems. *Chemosphere* 184 53–61. 10.1016/j.chemosphere.2017.05.149 28578196

[B29] Joensen K. G. Scheutz F. Lund O. Hasman H. Kaas R. S. Nielsen E. M. (2014). Real-time whole-genome sequencing for routine typing, surveillance, and outbreak detection of verotoxigenic *Escherichia coli*. *J. Clin. Microbiol.* 52 1501–1510. 10.1128/JCM.03617-13 24574290 PMC3993690

[B30] Joensen K. G. Tetzschner A. M. Iguchi A. Aarestrup F. M. Scheutz F. (2015). Rapid and easy *in silico* serotyping of *Escherichia coli* isolates by use of whole-genome sequencing data. *J. Clin. Microbiol.* 53 2410–2426. 10.1128/JCM.00008-15 25972421 PMC4508402

[B31] Kapmaz M. Erdem F. Abulaila A. Yeniaras E. Oncul O. Aktas Z. (2016). First detection of NDM-1 with CTX-M-9, TEM, SHV and rmtC in *Escherichia coli* ST471 carrying IncI2, A/C and Y plasmids from clinical isolates in Turkey. *J. Glob. Antimicrob. Resist.* 7 152–153. 10.1016/j.jgar.2016.10.001 27835842

[B32] Larsen M. V. Cosentino S. Rasmussen S. Friis C. Hasman H. Marvig R. L. (2012). Multilocus Sequence Typing of total genome sequenced bacteria. *J. Clin. Micobiol.* 50 1355–1361. 10.1128/JCM.06094-11 22238442 PMC3318499

[B33] Lee H. Y. Chang R. C. Su L. H. Liu S. Y. Wu S. R. Chuang C. H. (2012). Wide spread of Tn2006 in an AbaR4-type resistance island among carbapenem-resistant *Acinetobacter baumannii* clinical isolates in Taiwan. *Int. J. Antimicrob. Agents* 40 163–167. 10.1016/j.ijantimicag.2012.04.018 22743015

[B34] Lima-Bittencourt C. I. Costa P. S. Barbosa F. A. Chartone-Souza E. Nascimento A. M. (2011). Characterization of a *Chromobacterium haemolyticum* population from a natural tropical lake. *Lett. Appl. Microbiol.* 52 642–650. 10.1111/j.1472-765X.2011.03052.x 21466570

[B35] Marti E. Variatza E. Balcazar J. L. (2014). The role of aquatic ecosystems as reservoirs of antibiotic resistance. *Trends Microbiol.* 22 36–41. 10.1016/j.tim.2013.11.001 24289955

[B36] Mattos K. P. H. Visacri M. B. Quintanilha J. C. F. Lloret G. R. Cursino M. A. Levin A. S. (2017). Brazil’s resolutions to regulate the sale of antibiotics: impact on consumption and *Escherichia coli* resistance rates. *J. Glob. Antimicrob. Resist.* 10 195–199. 10.1016/j.jgar.2017.05.023 28735057

[B37] McArthur A. G. Waglechner N. Nizam F. Yan A. Azad M. A. Baylay A. J. (2013). The comprehensive antibiotic resistance database. *Antimicrob. Agents Chemother.* 57 3348–3357. 10.1128/AAC.00419-13 23650175 PMC3697360

[B38] Medeiros M. Lincopan N. (2013). Oxacillinase (OXA)-producing *Acinetobacter baumannii* in Brazil: clinical and environmental impact and therapeutic options. *J. Bras. Patol. Med. Lab.* 49 391–405. 10.1590/S1676-24442013000600003

[B39] Moura A. Oliveira C. Henriques I. Smalla K. Correia A. (2012). Broad diversity of conjugative plasmids in integron-carrying bacteria from wastewater environments. *FEMS Microbiol. Lett.* 330 157–164. 10.1111/j.1574-6968.2012.02544.x 22409355

[B40] Nascimento T. Cantamessa R. Melo L. Lincopan N. Fernandes M. R. Cerdeira L. (2017). International high-risk clones of *Klebsiella pneumoniae* KPC-2/CC258 and *Escherichia coli* CTX-M-15/CC10 in urban lake waters. *Sci. Total Environ.* 598 910–915. 10.1016/j.scitotenv.2017.03.207 28458208

[B41] Overbeek R. Olson R. Pusch G. D. Olsen G. J. Davis J. J. Disz T. (2014). The SEED and the Rapid annotation of microbial genomes using subsystems technology (RAST). *Nucleic Acids Res.* 42 D206–D214. 10.1093/nar/gkt1226 24293654 PMC3965101

[B42] Pei R. Gunsch C. K. (2009). Plasmid conjugation in an activated sludge microbial community. *Environ. Eng. Sci.* 26 825–831. 10.1089/ees.2008.0236

[B43] Peirano G. Asensi M. D. Pitondo-Silva A. Pitout J. D. D. (2011). Molecular characteristics of extended-spectrum beta-lactamase-producing *Escherichia coli* from Rio de Janeiro. *Brazil. Clin. Microbiol. Infect.* 17 1039–1043. 10.1111/j.1469-0691.2010.03440.x 21722255

[B44] Poirel L. Kampfer P. Nordmann P. (2002). Chromosome-encoded Ambler class A beta-lactamase of Kluyvera georgiana a probable progenitor of a subgroup of CTX-M extended-spectrum beta-lactamases. *Antimicrob. Agents Chemother.* 46:4038. 10.1128/AAC.46.12.4038-4040.2002 12435721 PMC132763

[B45] Rice E. W. Baird R. B. Eaton A. D. Clesceri L. S. (2012). *Standard Methods for the Examination of Water and Wastewater.* Washington, DC: APHA.

[B46] Rocha F. R. Pinto V. P. T. Barbosa F. C. B. (2016). The spread of CTX-M-type Extended-Spectrum beta-Lactamases in Brazil: a systematic review. *Microb. Drug Resist.* 22 301–311. 10.1089/mdr.2015.0180 26669767

[B47] Rosewarne C. P. Pettigrove V. Stokes H. W. Parsons Y. M. (2010). Class 1 integrons in benthic bacterial communities: abundance, association with Tn402-like transposition modules and evidence for coselection with heavy metal resistance. *FEMS Microbiol. Ecol.* 72 35–46. 10.1111/j.1574-6941.2009.00823.x 20132306

[B48] Rutherford K. Parkhill J. Crook J. Horsnell T. Rice P. Rajandream M. A. (2000). Artemis: sequence visualization and annotation. *Bioinformatics* 16 944–945. 11120685 10.1093/bioinformatics/16.10.944

[B49] Santos M. L. S. Saraiva A. L. L. Pereira J. A. R. Nogueira P. F. S. M. Silva A. C. (2015). Hydrodynamic modelling of a reservoir used to supply water to Belém (Lake Agua Preta. Para, Brazil). *Acta Sci. Technol.* 37 353–359. 10.4025/actascitechnol.v37i3.25839

[B50] Sellera F. P. Fernandes M. R. Moura Q. Souza T. A. Cerdeira L. Lincopan N. (2017). Draft genome sequence of *Enterobacter cloacae* ST520 harbouring blaKPC-2, blaCTX-M-15 and blaOXA-17 isolated from coastal waters of the South Atlantic Ocean. *J. Glob. Antimicrob. Resist.* 10 279–280. 10.1016/j.jgar.2017.07.017 28827199

[B51] Shepard S. M. Danzeisen J. L. Isaacson R. E. Seemann T. Achtman M. Johnson T. J. (2012). Genome sequences and phylogenetic analysis of K88- and F18-positive porcine enterotoxigenic *Escherichia coli*. *J. Bacteriol.* 194 395–405. 10.1128/JB.06225-11 22081385 PMC3256668

[B52] Soares S. C. Geyik H. Ramos R. T. de Sá P. H. Barbosa E. G. Baumbach J. (2016). GIPSy: genomic island prediction software. *J. Biotechnol.* 232 2–11. 10.1016/j.jbiotec.2015.09.008 26376473

[B53] Tacão M. Araújo S. Vendas M. Alves A. Henriques I. (2018). *Shewanella* species as the origin of *bla*OXA-48 genes: insights into gene diversity, associated phenotypes and possible transfer mechanisms. *Int. J. Antimicrob. Agents.* 51 340–348. 10.1016/j.ijantimicag.2017.05.014 28666748

[B54] Tacão M. Correia A. Henriques I. (2012). Resistance to broad-spectrum antibiotics in aquatic systems: anthropogenic activities modulate the dissemination of blaCTX-M-like genes. *App. Environ. Microbiol.* 78 4134–4140. 10.1128/AEM.00359-12 22492443 PMC3370516

[B55] Tacão M. Moura A. Correia A. Henriques I. (2014). Co-resistance to different classes of antibiotics among ESBL-producers from aquatic systems. *Water Res.* 48 100–107. 10.1016/j.watres.2013.09.021 24091187

[B56] Taylor N. G. Verner-Jeffreys D. W. Baker-Austin C. (2011). Aquatic systems: maintaining, mixing and mobilising antimicrobial resistance? *Trends Ecol. Evol.* 26 278–284. 10.1016/j.tree.2011.03.004 21458879

[B57] Tian S. F. Chu Y. Z. Chen B. Nian H. Shang H. (2011). ISEcp1 element in association with *bla*CTX-M genes of *E. coli* that produce extended-spectrum beta-lactamase among the elderly in community settings. *Enferm. Infecc. Microbiol. Clin.* 29 731–734. 10.1016/j.eimc.2011.07.011 22019175

[B58] Toleman M. A. Bennett P. M. Walsh T. R. (2006). ISCR elements: novel gene-capturing systems of the 21st century? *Microbiol. Mol. Biol. Rev.* 70 296–316. 16760305 10.1128/MMBR.00048-05 PMC1489542

[B59] Versalovic J. Schneider M. De Bruijn F. J. Lupski J. R. (1994). Genomic fingerprinting of bacteria using repetitive sequence-based polymerase chain reaction. *Methods Mol. Cell. Biol.* 5 25–40.

[B60] WHO (2014). *Antimicrobial Resistance: Global Report on Surveillance.* Geneva: WHO.

[B61] Wright M. S. Baker-Austin C. Lindell A. H. Stepanauskas R. Stokes H. W. McArthur J. V. (2008). Influence of industrial contamination on mobile genetic elements: class 1 integron abundance and gene cassette structure in aquatic bacterial communities. *ISME J.* 2 417–428. 10.1038/ismej.2008.8 18273063

[B62] Yi J. Kim N. Ko M. K. Kim H. Kim S. R. Hong S. H. (2017). Epidemiological and molecular characteristics of carbapenemase-producing *Enterobacteriaceae* in a tertiary hospital in korea: possible emergence of KPC-producing *Escherichia coli* ST471 strain. *Open Forum Infect. Dis.* 4:S599 10.1093/ofid/ofx163.1574

[B63] Zankari E. Hasman H. Cosentino S. Vestergaard M. Rasmussen S. Lund O. (2012). Identification of acquired antimicrobial resistance genes. *J. Antimicrob. Chemother.* 67 2640–2644. 10.1093/jac/dks261 22782487 PMC3468078

[B64] Zhao W. H. Hu Z. Q. (2012). *Acinetobacter*: a potential reservoir and dispenser for beta-lactamases. *Crit. Rev. Microbiol.* 38 30–51. 10.3109/1040841X.2011.621064 22007992

[B65] Zhong Z. Lu X. Valenzuela J. K. Partridge S. R. Iredell J. (2008). An outbreak of carbapenem-resistant *Acinetobacter baumannii* producing OXA-23 carbapenemase in western China. *Int. J. Antimicrob. Agents* 31 50–54. 18077141 10.1016/j.ijantimicag.2007.08.019

[B66] Zurfluh K. Hächler H. Nüesch-Inderbinen M. Stephan R. (2013). Characteristics of extended-spectrum β-lactamase- and carbapenemase-producing *Enterobacteriaceae* Isolates from rivers and lakes in Switzerland. *Appl. Environ. Microbiol.* 79 3021–3026. 10.1128/AEM.00054-13 23455339 PMC3623138

[B67] Zurfluh K. Tasara T. Stephan R. (2016). Full-genome sequence of *Escherichia coli* K-15K-W01, a uropathogenic E. *coli B*2 sequence type 127 isolate harboring a chromosomally carried blaCTX-M-15 gene. *Genome Announc.* 4:e00927-16. 10.1128/genomeA.00927-16 PMC5009988 27587831

