# Supplementary material for: Extended Spectrum Beta-Lactamase-Producing Gram-Negative Bacteria Recovered From an Amazonian Lake Near the City of Belém, Brazil
Source: Front Microbiol. 2019 Feb 28;10:364. doi: 10.3389/fmicb.2019.00364 (PMC6403167; doi:10.3389/fmicb.2019.00364)
Supplement: Supplementary file 1 [file Data_Sheet_1.docx]

**SUPPLEMENTARY MATERIAL**

**Figure S1. M**etal-resistance operons of *Escherichia coli* APC43A and *Acinetobacter baumannii* APC25. Gene synteny was analyzed in the web-based platform Simple Synteny. Each jagged edge contains the position in base pairs of each operon detected. A 50% identity was used to find genes in the genomes. The absence of lines connecting the genes demonstrates that each genome has distinct operons to metal resistance.

**
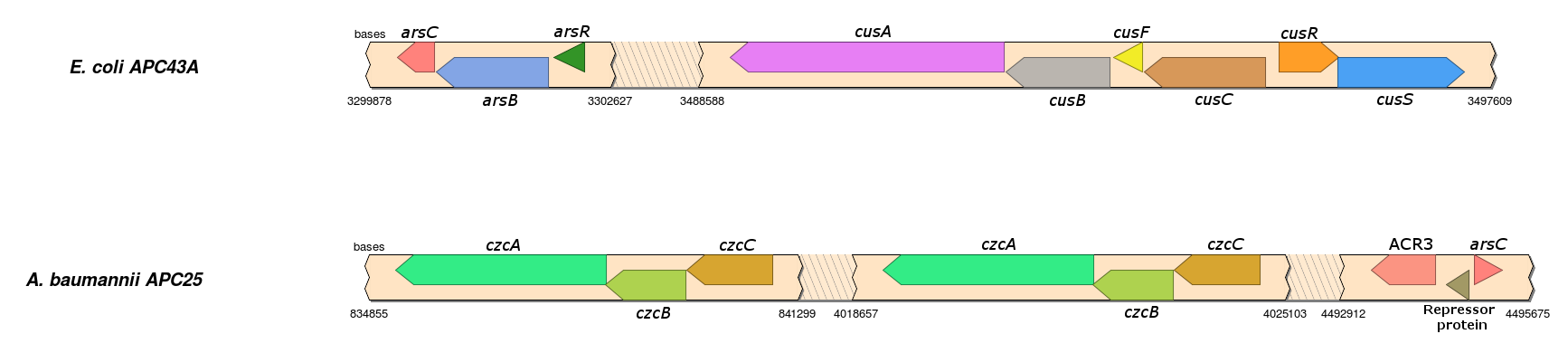
**

**Table S1.** Physical, chemical and microbiological parameters of water collected at the six sampling points in Lake Água Preta (map presented in Figure 1). Each sampling point was analyzed in triplicate and the numbers in the table represent the mean values. Highlighted in red are the parameters that were not in accordance with the Brazilian law.

| **Parameters** | **S1** | **S2** | **S3** | **S4** | **S5** | **S6** |
| --- | --- | --- | --- | --- | --- | --- |
| Latitude | 01°21’28.5’’ | 01°24’17.2’’ | 01°25’20.7’’ | 01°25’39.8’’ | 01°24’31.6’’ | 01°24’32.2’’ |
| Longitude | 48°25’15.1’’ | 48°24’55.8’’ | 48°25’04.3’’ | 48°24’37.7’’ | 48°24’07.9’’ | 48°24’19.7’’ |
| pH | 6,2 | 5 | 5,2 | 4,8 | 5,4 | 5,1 |
| Temperature (°C) | 34,5 | 32 | 33,8 | 31,2 | 32,6 | 32,8 |
| DO (mg O_2_.L^-1^) | 5 | 4,5 | 4,9 | 4,6 | 4,2 | 4,3 |
| Conductivity (µS.cm^-1^) | 30 | 45 | 23 | 38 | 57 | 63 |
| Total solids (mg.L^-1^) | 118 | 120 | 310 | 172 | 80 | 82 |
| Apparent color (UPtCo.L^-1^) | 83 | 62 | 112 | 122 | 39 | 44 |
| True color (UPtCo.L^-1^) | 47 | 43 | 68 | 57 | 28 | 27 |
| Total N (mg N.L^-1^) | 7,65 | 9,88 | 13,95 | 13,7 | 3,74 | 14,2 |
| Ammonia (mg N-NH_3_.L^-1^) | 1,09 | 0,31 | 0,05 | 1,0 | 0,70 | 1,7 |
| Nitrite (mg N-NO^2-^.L^-1^) | 0,03 | 0,06 | 0,03 | 0,6 | 0,52 | 0,01 |
| Nitrate (mg N-NO^3-^.L^-1^) | 6,3 | 9,1 | 13 | 12 | 2,3 | 12,3 |
| Turbity (UNT) | 15 | 9 | 28 | 53 | 7 | 6 |
| Total P (mg P.L^-1^) | 0,16 | 0,4 | 0,04 | 0,04 | 0,08 | 0,88 |
| Odor (Intensity) | Absent (0) | Absent (0) | Absent (0) | Absent (0) | Absent (0) | Absent (0) |
| Total alkalinity (mg CaCO_3_.L^-1^) | 20 | 34 | 14 | 10 | 34 | 24 |
| Acidity (mg CaCO_3_.L^-1^) | 18 | 12 | 11 | 8 | 19 | 11 |
| Chlorides (mg Cl^-^.L^-1^) | 11 | 71 | 45 | 2,4 | 8 | 3,2 |
| Aluminum (mg Al.L^-1^) | 2,95 | 1,6 | 2,96 | 3,81 | 0,31 | 0,48 |
| Sulfate (mg.L^-1^) | < DL^a^ | <DL^a^ | <DL^a^ | <DL^a^ | <DL^a^ | <DL^a^ |
| Total iron (mg.L^-1^) | 1,58 | 1,02 | 2,21 | 2,86 | 0,60 | 0,73 |
| Manganese (mg.L^-1^) | 0,035 | 0,02 | 0,04 | 0,05 | 0,02 | 0,03 |
| Nickel (mg.L^-1^) | <DL^a^ | <DL^a^ | 0,007 | <DL^a^ | <DL^a^ | <DL^a^ |
| Cadmium (mg.L^-1^) | <DL^a^ | <DL^a^ | <DL^a^ | <DL^a^ | <DL^a^ | <DL^a^ |
| Copper (mg.L^-1^) | <DL^a^ | 0,002 | 0,004 | <DL^a^ | <DL^a^ | <DL^a^ |
| Zinc (mg.L^-1^) | 0,005 | <DL^a^ | 0,015 | 0,002 | 0,001 | <DL^a^ |
| COD (mg O_2_.L^-1^) | 12 | 10 | 18 | 18 | 12 | 12 |
| BOD (mg O_2_.L^-1^) | 4 | <DL^a^ | <DL^a^ | 7 | 6 | 5 |
| Total coliforms (MPN 100 mL^-1^) | 1,1x10^3^ | 0,9x10^3^ | 0,5x10^3^ | 0,3x10^3^ | 0,6x10^3^ | 0,7x10^3^ |
| Thermotolerant coliforms (MPN 100 mL^-1^) | 272 | 141 | 141 | 86 | 36 | 108 |

^a^ Below the limit of detection.

**Table S2.** Minimal inhibitory concentrations (MIC) for the 3^rd^ generation cephalosporins cefotaxime (CTX) and ceftazidime (CAZ), of *bla*_CTX-M_-carrying isolates and transconjugants.

| ***bla*_CTX-M_-carrying donor strains** | | | ***bla*_CTX-M_-carrying transconjugants** | |
| --- | --- | --- | --- | --- |
| Donors | MIC µg/mL (R/I/S) * | | MIC µg/mL (R/I/S) * | |
|  | CTX | CAZ | CTX | CAZ |
| **APC43A** | >256 (R) | 4 (S) | - |  |
| **APC22** | >256 (R) | 2 (S) | - |  |
| **APC24B** | 4 | 8 (I) | - |  |
| **APC28** | 64(R) | 8 (I) | >256 (R) | 8 (I) |
| **APC32** | >256 (R) | 8 (I) | >256 (R) | 8 (I) |
| **APC33** | >256 (R) | 8 (I) | - | - |
| **APC34** | 32 (R) | 16 (R) | - | - |
| **APC40A** | >256 (R) | 2 (S) | >256 (R) | 4 (S) |
| **APC43B** | >256 (R) | 8 (I) | >32 (R) | 4 (S) |
| **Recipient Strain *E. coli* CV601** | 0.06 (S) | < 0.25 (S) |  |  |

*R – resistant, I – intermediate, S – susceptible

Table S3. Functional classes of predicted genes according to RAST annotation for *Escherichia coli* APC43A*.*

| **Functional Class** | **Genomic features** | **Genes (%)** |
| --- | --- | --- |
| Cofactors, Vitamins, Prosthetic Groups, Pigments | 327 | 6.4 |
| Cell Wall and Capsule | 322 | 6.3 |
| Virulence, Disease and Defense | 162 | 3.2 |
| Potassium metabolism | 48 | 0.9 |
| Photosynthesis | 0 | 0.0 |
| Miscellaneous | 76 | 1.5 |
| Phages, Prophages, Transposable elements, Plasmids | 82 | 1.6 |
| Membrane Transport | 331 | 6.4 |
| Iron acquisition and metabolism | 27 | 0.5 |
| RNA Metabolism | 285 | 5.5 |
| Nucleosides and Nucleotides | 179 | 3.5 |
| Protein Metabolism | 356 | 6.9 |
| Cell Division and Cell Cycle | 52 | 1.0 |
| Motility and Chemotaxis | 95 | 1.8 |
| Regulation and Cell signaling | 223 | 4.3 |
| Secondary Metabolism | 35 | 0.7 |
| DNA Metabolism | 186 | 3.6 |
| Fatty Acids, Lipids, and Isoprenoids | 170 | 3.3 |
| Nitrogen Metabolism | 93 | 1.8 |
| Dormancy and Sporulation | 7 | 0.1 |
| Respiration | 211 | 4.1 |
| Stress Response | 228 | 4.4 |
| Metabolism of Aromatic Compounds | 33 | 0.6 |
| Amino Acids and Derivatives | 513 | 10.0 |
| Sulfur Metabolism | 62 | 1.2 |
| Phosphorus Metabolism | 68 | 1.3 |
| Carbohydrates | 967 | 18.8 |
| **TOTAL** | **8382** | **100.0** |

Table S4. Functional classes of predicted genes according to RAST annotation for *Acinetobacter baumannii* APC25*.*

| **Functional Class** | **Genomic features** | **Genes (%)** |
| --- | --- | --- |
| Cofactors, Vitamins, Prosthetic Groups, Pigments | 388 | 9.3 |
| Cell Wall and Capsule | 172 | 4.1 |
| Virulence, Disease and Defense | 109 | 2.6 |
| Potassium metabolism | 39 | 0.9 |
| Photosynthesis | 0 | 0.0 |
| Miscellaneous | 72 | 1.7 |
| Phages, Prophages, Transposable elements, Plasmids | 20 | 0.5 |
| Membrane Transport | 128 | 3.1 |
| Iron acquisition and metabolism | 28 | 0.7 |
| RNA Metabolism | 286 | 6.9 |
| Nucleosides and Nucleotides | 138 | 3.3 |
| Protein Metabolism | 369 | 8.9 |
| Cell Division and Cell Cycle | 52 | 1.3 |
| Motility and Chemotaxis | 0 | 0.0 |
| Regulation and Cell signaling | 127 | 3.1 |
| Secondary Metabolism | 14 | 0.3 |
| DNA Metabolism | 122 | 2.9 |
| Fatty Acids, Lipids, and Isoprenoids | 247 | 5.9 |
| Nitrogen Metabolism | 42 | 1.0 |
| Dormancy and Sporulation | 3 | 0.1 |
| Respiration | 172 | 4.1 |
| Stress Response | 160 | 3.8 |
| Metabolism of Aromatic Compounds | 131 | 3.2 |
| Amino Acids and Derivatives | 645 | 15.5 |
| Sulfur Metabolism | 83 | 2.0 |
| Phosphorus Metabolism | 67 | 1.6 |
| Carbohydrates | 543 | 13.1 |
| **TOTAL** | **4157** | **100.0** |

Table S5. PathogenFinder results.

| ***Escherichia coli* APC43A** | |
| --- | --- |
| Probability of being a human pathogen | 0.932 |
| Matches | 548 |
| Genome Coverage (%) | 9.82 |
| Pathogenic Families Matched | 542 |
| Non-Pathogenic Families Matched | 6 |
| The organisms is predicted as human pathogenic | Yes |
| ***Acinetobacter baumanii* APC25** | |
| Probability of being human pathogen | 0.317 |
| Matches | 8 |
| Genome Coverage (%) | 0.16 |
| Pathogenic Families Matched | 0 |
| Non-Pathogenic Families Matched | 8 |
| The organisms is predicted as human pathogenic | No |
